# Both Specific Endothelial and Proximal Tubular Adam17 Deletion Protect against Diabetic Nephropathy

**DOI:** 10.3390/ijms22115520

**Published:** 2021-05-24

**Authors:** Vanesa Palau, Bramasta Nugraha, David Benito, Julio Pascual, Maximilian Y. Emmert, Simon P. Hoerstrup, Marta Riera, Maria José Soler

**Affiliations:** 1 Department of Nephrology, Hospital del Mar-Institut Hospital del Mar d’Investigacions Mèdiques, 08003 Barcelona, Spain; vpalau@imim.es (V.P.); david.benito.guasch@gmail.com (D.B.); julpascual@gmail.com (J.P.); 2 Institute for Regenerative Medicine (IREM), Faculty of Medicine, University of Zurich, 8952 Schlieren, Switzerland; maximilian.emmert@access.uzh.ch (M.Y.E.); simon.hoerstrup@irem.uzh.ch (S.P.H.); 3 Institute of Parasitology, Vetsuisse Faculty and Faculty of Medicine, University of Zurich, CH-8057 Zurich, Switzerland; bramasta.nugraha@astrazeneca.com; 4 Department of Cardiovascular Surgery, Charité Universitätsmedizin Berlin, 10117 Berlin, Germany; 5 German Heart Center Berlin, Department of Cardiothoracic and Vascular Surgery, 13353 Berlin, Germany

**Keywords:** ADAM17, diabetic nephropathy, endothelial deletion, renal proximal tubular deletion, 3D proximal tubular spheroids

## Abstract

ADAM17 is a disintegrin and metalloproteinase capable of cleaving the ectodomains of a diverse variety of molecules including TNF-α, TGF-α, L-selectin, and ACE2. We have previously demonstrated that renal ADAM17 is upregulated in diabetic mice. The role of endothelial (*eAdam17*) and proximal tubular (*tAdam17*) *Adam17* deletion in renal histology, modulation of the renin angiotensin system (RAS), renal inflammation, and fibrosis was studied in a mouse model of type 1 Diabetes Mellitus. Moreover, the effect of *Adam17* deletion in an in vitro 3D cell culture from human proximal tubular cells under high glucose conditions was evaluated. *eAdam17* deletion attenuates renal fibrosis and inflammation, whereas *tAdam17* deletion decreases podocyte loss, attenuates the RAS, and decreases macrophage infiltration, α-SMA and collagen accumulation. The 3D in vitro cell culture reinforced the findings obtained in *tAdam17KO* mice with decreased fibrosis in the *Adam17* knockout spheroids. In conclusion, *Adam17* deletion either in the endothelial or the tubular cells mitigates kidney injury in the diabetic mice by targeting different pathways. The manipulation of *Adam17* should be considered as a therapeutic strategy for treating DN.

## 1. Introduction

Diabetic nephropathy (DN) is the leading cause of end-stage renal disease in developed countries. Early DN is associated with glomerular injury, hyperfiltration and microalbuminuria, both attributable to podocyte loss, and tubular and endothelial dysfunction [1,2]. DN progression is followed by proteinuria, extracellular matrix deposition, glomerulosclerosis, and tubulointerstitial fibrosis [3,4,5]. Macrophage infiltration in DN characteristically appears in both human and experimental models and it is associated with hypertrophy, hypercellularity, interstitial myofibroblast accumulation, and collagen-IV deposition in the glomeruli and tubulointerstitium [6]. Profibrotic growth factors, such as transforming growth factor β (TGF-β), have a key role in extracellular matrix accumulation in high-glucose environments [7,8].

The disintegrin and metalloproteinase domain 17 (ADAM17) has been shown to be upregulated in kidneys of diabetic mice, contributing to renal inflammation and fibrosis [9,10,11]. In vitro experiments mimicking the diabetic milieu demonstrated an increase in ADAM17 protein expression in podocytes, glomerular endothelial cells, mesangial cells, and tubular epithelial cells [12,13,14]. ADAM17 activity is involved in the shedding of EGFR ligands, inducing TGF-β upregulation and extracellular matrix accumulation [12,15].

*Adam17* knockout (*Adam17KO*) mice presented reduced renal fibrosis and inflammation after acute kidney injury (AKI) induction or angiotensin II (ANG II) infusion [16,17]. Furthermore, the decrease in cleaved tumor necrosis factor alpha (TNF-α) in these mice correlated with renal reduction in pro-inflammatory markers and infiltrating macrophages and neutrophils [16]. ADAM17 expression is increased in endothelial and proximal tubular cells of DN patients [18]. However, the effect of the specific deletion of endothelial or tubular *Adam17* on DN has not been previously studied. Here, we elucidate whether specific *Adam17* deletion on endothelial or renal proximal tubular cells has a beneficial effect in type 1 diabetic mice. Moreover, we performed an in vitro 3D-cell culture of human proximal tubular cells incubated with high-glucose as a disease model for kidney fibrosis aimed to decipher the effect of *Adam17* deletion against a fibrotic diabetic milieu.

## 2. Results

### 2.1. Confirmation of Adam17 Deletion on Endothelial and Renal Proximal Tubular Cells

*eAdam17* and *tAdam17* deletions were confirmed by immunohistochemistry, β-galactosidase staining and *Adam17* gene expression. *eAdam17KO* mice presented reduced ADAM17 expression in the endothelium of the kidney arteries as compared with wild-type mice. Within the kidney tubules, *tAdam17KO* mice presented reduced ADAM17 protein expression in the proximal tubular cells compared with wild-type mice (Supplementary Figure S1B).

In addition, β-galactosidase staining as control of recombination was performed. *tAdam17* mice presented the ROSA26-driven LacZ-Cre reporter with β-galactosidase activity after a STOP codon flanked by loxP sites (Figure S1C). Therefore, only if recombination takes place will positive staining for β-galactosidase be detected. Moreover, we also showed lower *Adam17* gene expression in *tAdam17KO* mice as compared to WT mice (Supplementary Figure S1D).

### 2.2. Tubular Adam17 Knockout Protects from DN Hyperfiltration

Increased blood glucose levels and decreased body weight (BW) were observed in all diabetic mice. Kidney hypertrophy was calculated by the ratio kidney weight (KW)/BW. Diabetic animals presented a higher KW/BW ratio than their controls in both animal models. Diabetic tAdam17KO mice presented a lower KW/BW ratio when compared with diabetic wild-type mice, suggesting a reduced hypertrophy (Table 1).

Diabetes was associated with increased urine albumin-to-creatinine ratio in both animal models (Supplementary Figure S2).

### 2.3. Diabetic Kidneys of Adam17-Deficient Mice at Either Proximal Tubular Cells or Endothelial Cells Are Protected from Matrix Mesangial Expansion

**Figure 1:**
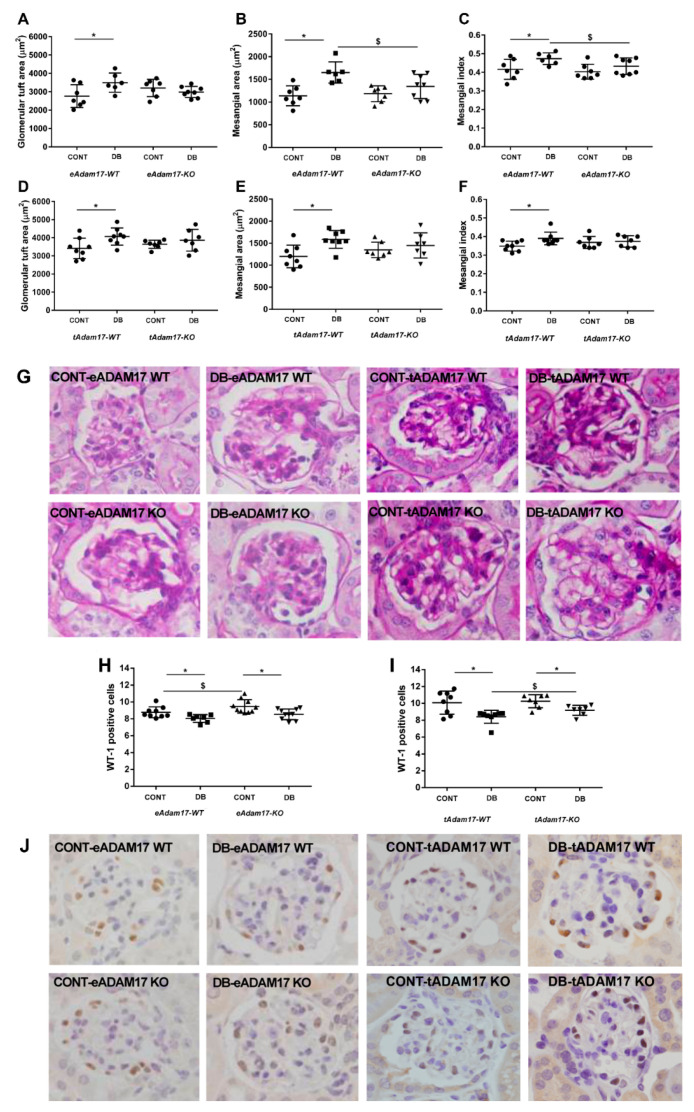
Glomerular tuft area, mesangial area, mesangial index and podocyte loss. (**A**) Glomerular tuft area from *eAdam17* model. (**B**) Mesangial expansion from *eAdam17* model. (**C**) Mesangial index from *eAdam17* model. (**D**) Glomerular tuft area from *tAdam17* model. (**E**) Mesangial expansion from *tAdam17* model. (**F**) Mesangial index from *tAdam17* model. (**G**) Representative images of the PAS staining for *eAdam17* and *tAdam17* mice. Original magnification, ×400. (**H**) Podocyte number is represented as the number of brown positive cells after WT-1 immunostaining in *eAdam17* model. (**I**) Podocyte number is represented as the number of brown positive cells after WT-1 immunostaining in *tAdam17* model. (**J**) Representative images of the WT-1 immunostaining for *eAdam17* and *tAdam17* mice. Original magnification, ×400. CONT, control; DB, diabetic; eAdam17, endothelial ADAM17; KO, knockout; tAdam17, proximal tubular ADAM17; WT, wild-type; WT-1, wilms tumor 1. * *p* < 0.05 DB vs. NoDB, $ *p* < 0.05 KO vs. WT; n = 6–9 in each group.

PAS-stained kidney samples revealed a significant increase in glomerular tuft area, matrix mesangial expansion and mesangial index in all diabetic wild-type mice. This increase was attenuated in diabetic knockout mice (Figure 1A–G). Moreover, among diabetic animals, *eAdam17KO* mice exhibited a significantly decreased mesangial matrix expansion and mesangial index when compared with wild-type mice (Figure 1B,C).

### 2.4. Adam17 Deletion in Proximal Tubular Cells Protects against Podocyte Loss

The podocyte number was significantly decreased in diabetic groups. However, diabetic *tAdam17KO* displayed lower podocyte loss in comparison with diabetic wild-type mice (Figure 1H–J).

### 2.5. Adam17 Deletion in Proximal Tubular Cells Attenuates the Renin Angiotensin System

**Figure 2:**
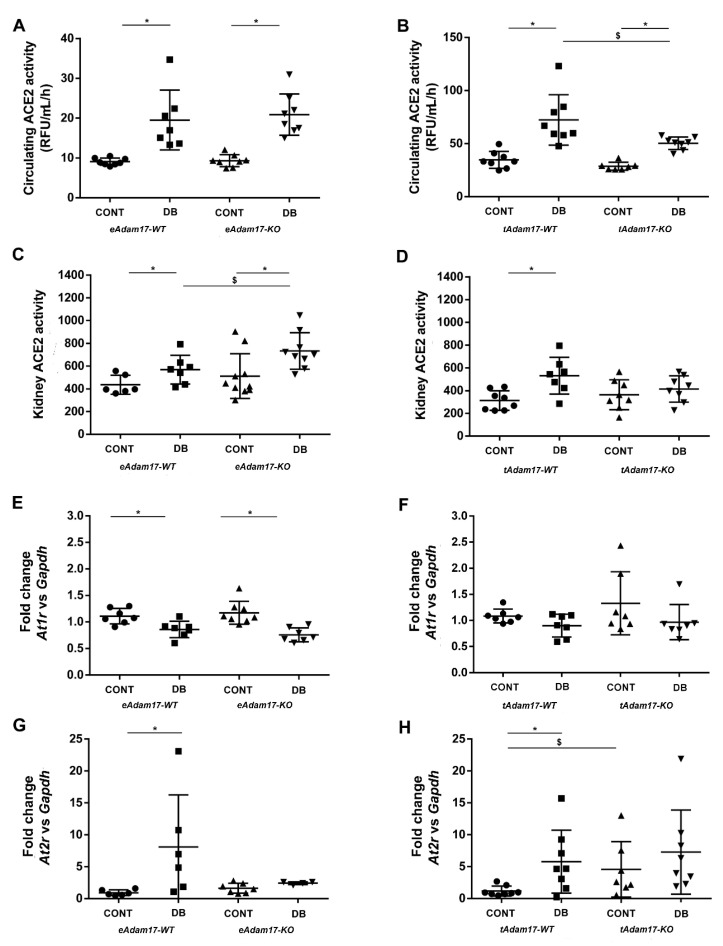
Effect of diabetes and endothelial or proximal-tubular *Adam17* deletion on the Renin Angiotensin System. (**A**) Circulating ACE2 activity from *eAdam17* model. (**B**) Circulating ACE2 activity from *tAdam17* model. (**C**) Renal ACE2 activity from *eAdam17* model. (**D**) Renal ACE2 activity from *tAdam17* model. (**E**) *At1r* gene expression from *eAdam17* model. (**F**) *At1r* gene expression from *tAdam17* model. (**G**) *At2r* gene expression from *eAdam17* model. (**H**) *At2r* gene expression from *tAdam17* model. ACE2, angiotensin converting enzyme 2; AT1R, angiotensin II receptor type 1; AT2R, angiotensin II receptor type 2; CONT, control; DB, diabetic; eAdam17, endothelial ADAM17; KO, knockout; tAdam17, proximal tubular ADAM17; WT, wild-type. * *p* < 0.05 DB vs. NoDB, $ *p* < 0.05 KO vs. WT; n = 6–8 in each group.

Circulating ACE2 enzymatic activity was increased in all diabetic mice. Interestingly, *tAdam17* deletion in diabetic mice reduced circulating ACE2 activity when compared with diabetic wild-type mice (Figure 2A,B).

Renal ACE2 enzymatic activity was significantly increased in diabetic mice (Figure 2C,D). *eAdam17* deletion exacerbates renal ACE2 activity in such mice (Figure 2C), while *tAdam17* deletion attenuates the increase in renal ACE2 activity in diabetic mice (Figure 2D).

We also analysed the gene expression of different RAS components. All diabetic mice presented upregulated *Agt* gene expression (Supplementary Figure S3). Regarding ANG II receptors, *At1r* gene expression was downregulated in *eAdam17* diabetic mice (Figure 2E). In contrast, *At2r* gene expression increased in diabetic wild-type mice of both animal models (Figure 2G,H).

### 2.6. Adam17 Deletion Decreased Inflammation in DN

**Figure 3:**
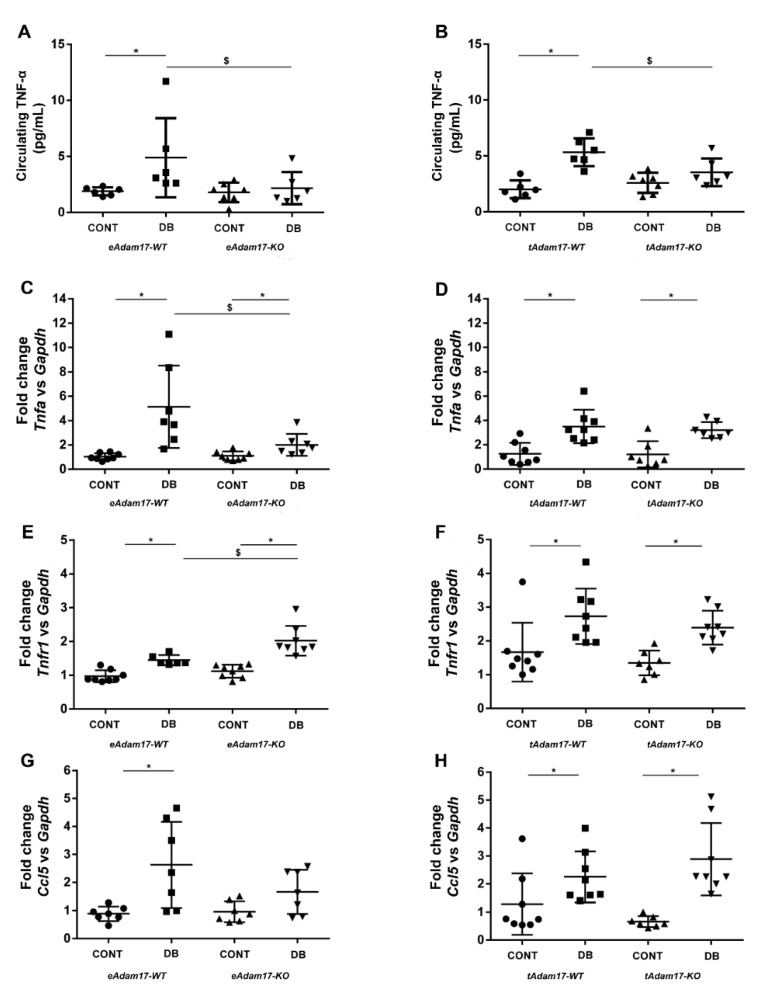
Influence of diabetes and *Adam17* deletion on circulating TNF-α, *Tnfa*, *Tnfr1*, and *Ccl5* gene expression. (**A**) Circulating TNF-α from *eAdam17* model. (**B**) Circulating TNF-α from *tAdam17* model. (**C**) *Tnfa* gene expression from *eAdam17* model. (**D**) *Tnfa* gene expression from *tAdam17* model. (**E**) *Tnfr1* gene expression from *eAdam17* model. (**F**) *Tnfr1* gene expression from *tAdam17* model. (**G**) *Ccl5* gene expression from *eAdam17* model. (**H**) *Ccl5* gene expression from *tAdam17* model. CONT, control; DB, diabetic; eAdam17, endothelial ADAM17; KO, knockout; tAdam17, proximal tubular ADAM17; TNF-α, tumor necrosis factor α; WT, wild-type. * *p* < 0.05 DB vs. NoDB, $ *p* < 0.05 KO vs. WT; n = 6–8 in each group.

To evaluate whether deleting *Adam17* in endothelial or proximal tubular cells reduces renal inflammation, we analysed the expression of different pro-inflammatory markers. Circulating TNF-α levels were significantly increased in diabetic wild-type mice in comparison with controls. Interestingly, among diabetic animals, both knockout mice exhibited significantly decreased TNF-α levels when compared with wild-type mice (Figure 3A,B). Moreover, hyperglycaemia was accompanied by a significant increase in mRNA levels of *Tnfα* (Figure 3C,D). Similarly, *Tnfr1* expression was upregulated in all diabetic mice (Figure 3E,F). Interestingly, diabetic *eAdam17KO* mice presented decreased *Tnf-α* (Figure 3C) and increased *Tnfr1* (Figure 3E) gene expression when compared with *eAdam17WT* mice.

A similar pattern was observed for chemokine *Ccl5* ((C-C motif) ligand 5) gene expression. While hyperglycaemia induced an increase in *Ccl5* gene expression, *Adam17* deletion in endothelial cells attenuates *Ccl5* upregulation in diabetic mice (Figure 3G).

**Figure 4:**
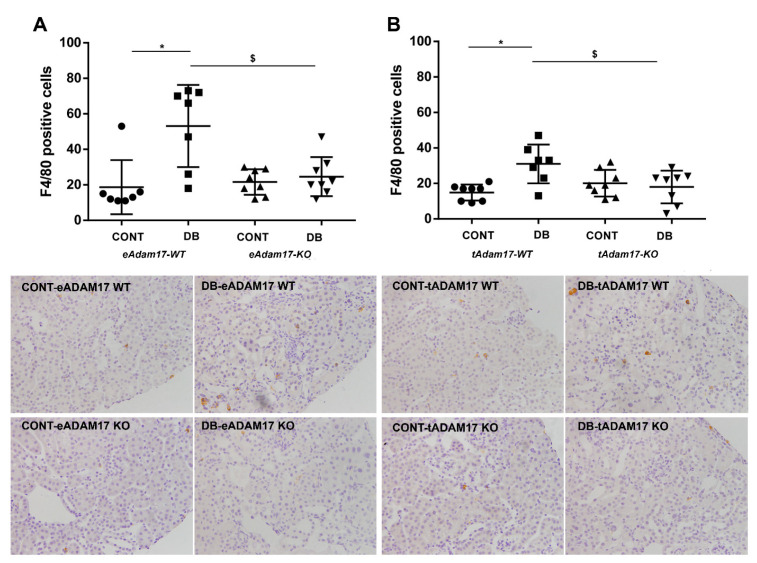
Influence of diabetes and *Adam17* deletion on F4/80 immunostaining. (**A**) F4/80 immunostaining from *eAdam17* model. (**B**) F4/80 immunostaining from *tAdam17* model. Original magnification, ×200. CONT, control; DB, diabetic; eAdam17, endothelial ADAM17; KO, knockout; tAdam17, proximal tubular ADAM17; TNF-α, tumor necrosis factor α; WT, wild-type. * *p* < 0.05 DB vs. NoDB, $ *p* < 0.05 KO vs. WT; n = 6–8 in each group.

Macrophage infiltration in the kidney was assessed by F4/80 protein detection. In both strains, F4/80 staining was increased in wild-type diabetic mice as compared to controls. *Adam17* deletion either in endothelial or proximal tubular cells reduced macrophage infiltration in diabetic mice in comparison with diabetic wild-type mice (Figure 4).

### 2.7. Diabetic Kidneys of Adam17 Deficient Mice in Either Proximal Tubular Cells or Endothelial Cells Are Protected from Renal Fibrosis

**Figure 5:**
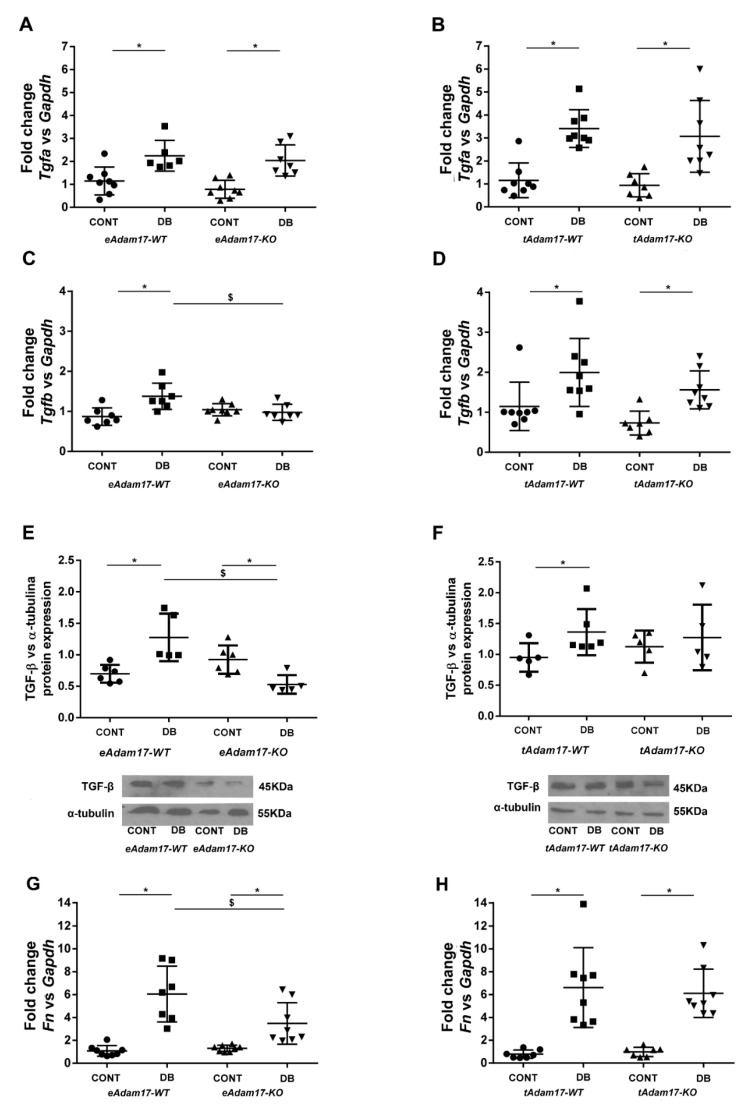
Influence of diabetes and *Adam17* deletion on *Tgfα*, *Tgfβ*, and *fibronectin* gene expression and protein expression. (**A**) *Tgfα* gene expression from the *eAdam17* model. (**B**) *Tgfα* gene expression from the *tAdam17* model. (**C**) *Tgfβ* gene expression from the *eAdam17* model. (**D**) *Tgfβ* gene expression from the *tAdam17* model. (**E**) TGF-β protein expression from *eAdam17* model. (**F**) TGF-β protein expression from *tAdam17* model. (**G**) *Fibronectin* gene expression from the *eAdam17* model. (**H**) *Fibronectin* gene expression from the *tAdam17* model. CONT, control; DB, diabetic; eAdam17, endothelial ADAM17; FN, fibronectin; KO, knockout; tAdam17, proximal tubular ADAM17; TGF-α, transforming growth factor α, TGF-β, transforming growth factor β; WT, wild-type. * *p* < 0.05 DB vs. NoDB, $ *p* < 0.05 KO vs. WT; n = 6–8 in each group.

To establish whether *Adam17* deletion ameliorates renal fibrosis, gene expression for different fibrotic markers related to EGFR signaling pathways was studied. *Tgfα* and *Hb-egf* gene expression were analysed as substrates of the EGFR signaling pathway. In both strains, *Tgfα* gene expression was increased in all diabetic mice (Figure 5A,B). However, no differences were observed between groups regarding *Hb-egf* gene expression (Supplementary Figure S4).

For downstream effectors of the EGFR signaling pathway, we observed downregulated *Tgfβ* gene expression in diabetic mice except for the *eAdam17KO* mice. Interestingly, only *eAdam17* deletion ameliorated the diabetes effect on renal *Tgfβ* gene expression (Figure 5C,D). At the protein level, only diabetic wild-type mice increased TGF-β (Figure 5E,F). Surprisingly, diabetic *eAdam17KO* mice presented lower TGF-β protein expression than control mice (Figure 5E). Moreover, both endothelial and proximal tubular *Adam17* deletion reduced or attenuated TGF-β production in diabetic mice.

An increase in *Fn* gene expression was seen in all diabetic mice and *Fn* gene expression was attenuated only in diabetic *eAdam17KO* mice (Figure 5).

**Figure 6:**
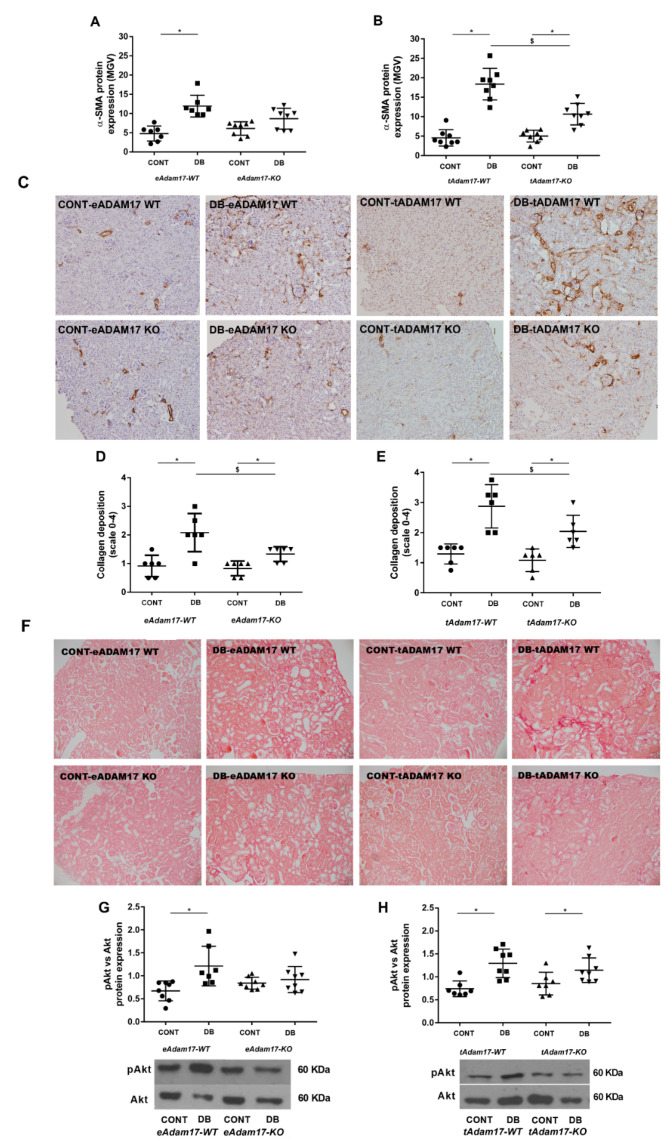
Influence of diabetes and *Adam17* deletion on α-SMA and collagen staining and pAkt/Akt ratio. (**A**) α-SMA immunostaining from *eAdam17* model. (**B**) α-SMA immunostaining from *tAdam17* model. (**C**) Representative images of α-SMA immunostaining from *eAdam17* and *tAdam17* models. Original magnification, ×100. (**D**) Collagen staining from *eAdam17* model. (**E**) Collagen staining from *tAdam17* model. (**F**) Representative images of Sirius Red staining from *eAdam17* and *tAdam17* models. Original magnification, ×100. (**G**) pAkt/Akt ratio from *eAdam17* model. (**H**) pAkt/Akt ratio from *tAdam17* model. CONT, control; DB, diabetic; eAdam17, endothelial ADAM17; KO, knockout; pAkt, phosphorylated protein kinase B; α-SMA, alpha smooth muscle actin; tAdam17, proximal tubular ADAM17; WT, wild-type. * *p* < 0.05 DB vs. NoDB, $ *p* < 0.05 KO vs. WT; n = 6–8 in each group.

To gain an insight into the role of *Adam17* deletion on renal fibrosis, we examined cortical α-SMA expression and collagen depositions in the studied groups. As depicted in Figure 5A–C, diabetes induced α-SMA accumulation in the interstitial compartment of both animal models. However, this effect was subtly lost on diabetic *eAdam17* knockout mice (Figure 6A).

In concordance with gene expression data, semiquantitative scoring of collagen deposition showed increased accumulation of collagen fibres in diabetic mice. In contrast to the gene expression profiles, collagen accumulation was downregulated by endothelial and proximal tubular *Adam17* deletion in diabetic mice (Figure 6D–F).

PI3K/Akt signaling pathway is the downstream mediator of the EGFR, important in glucose-induced pro-fibrotic responses in the kidney. While all diabetic mice exhibited a significantly increased pAkt/Akt ratio in renal tissue, *eAdam17* deletion attenuated diabetes’ effect on pAkt/Akt ratio (Figure 6).

### 2.8. Fibrotic Markers Expression in Adam17-Deleted HKC-8 Spheroids

To further delineate the effect of *tAdam17* deletion against fibrosis, a 3D in vitro culture with HKC-8 cells resembling the human diabetic microenvironment was used.

**Figure 7:**
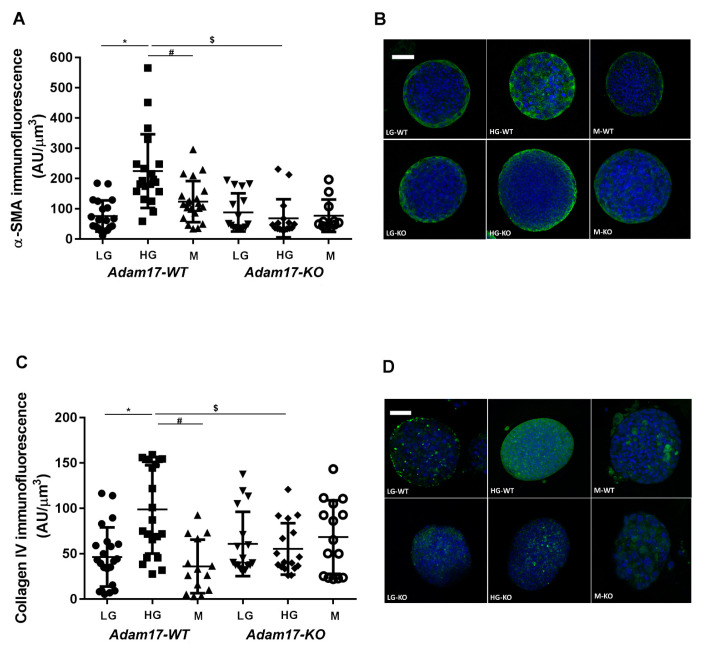
Influence of high glucose medium and *Adam17* deletion on α-SMA and Collagen-IV immunofluorescence staining. (**A**) α-SMA immunofluorescence staining in HKC-8 spheroids. (**B**) Representative images of the α-SMA immunofluorescence staining. (**C**) Type IV collagen immunofluorescence staining in HKC-8 spheroids. (**D**) Representative images of the Type IV collagen immunofluorescence staining. LG, low glucose; HG, high glucose; M, mannitol. * *p* < 0.05 HG vs. LG, $ *p* < 0.05 KO vs. WT, # *p* < 0.05 M vs. HG; n = 15–21 spheroids per group. Scale bar 20 µm.

Mature HKC-8 spheroids expressed proximal tubular transporters GLUT-1 and AQP1 keeping phenotypic characteristics (data not shown). In concordance with our results in mice, wild-type *Adam17* spheroids incubated with HG for 72 h increased the expression of α-SMA and type-IV collagen. Interestingly, *Adam17* deletion blocked the increase of α-SMA and type-IV collagen expression compared with wild-type cells (Figure 7A–D).

## 3. Discussion

**Figure 8:**
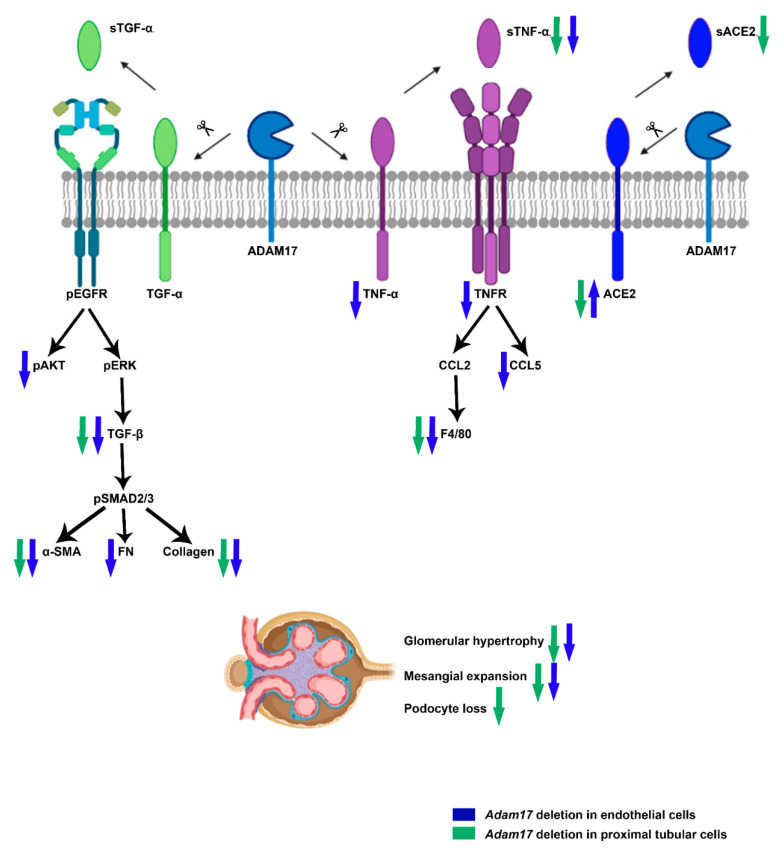
Schematic figure summarizing the findings regarding the effects of *Adam17* deletion in endothelial and proximal tubular cells under STZ-diabetic mice model.

We demonstrate that specific *Adam17* deletion on endothelial cells prevents renal pro-inflammatory and pro-fibrotic events caused by DN, and specific *Adam17* deletion on renal proximal tubular cells protect from diabetic pro-fibrotic events, podocyte loss, and attenuates renal RAS. Remarkably, this is the first evidence of the beneficial effect of *Adam17* downregulation either in endothelial or renal proximal tubular cells in experimental DN (Figure 8).

Hyperglycaemia stimulates cells to release pro-inflammatory cytokines into the kidney, leading to the recruitment of fibroblasts, which initiates fibrotic processes [19]. ADAM17 is a disintegrin and metalloprotease associated with the shedding of most pro-inflammatory and pro-fibrotic substrates [15,20,21]. Targeting the specific inflammatory and fibrotic pathways could be an effective approach for the management of DN.

Although paricalcitol, an analogue of the active form of vitamin D with ADAM17 inhibitory properties had shown a reduction in ACR levels in diabetic patients [22]; our study showed that *Adam17* deletion either in endothelial or renal proximal tubular cells was unable to prevent albuminuria in the STZ model. The lack of effect on albuminuria in *Adam17* knockout mice might be ascribed to the huge ACR variability among animals. This variability could be associated with the urine collection method by morning urine spots.

Regarding renal hypertrophy, we demonstrated that *tAdam17* deletion in diabetic mice reduced the KW/BW ratio as previously shown with paricalcitol administration in non-obese diabetic (NOD) mice [11]. Taken together, ADAM17 modulation may exert a positive effect in the diabetic kidney by decreasing renal hypertrophy.

At the glomerular level, our histological analyses revealed glomerular alterations in diabetic mice due to an increased mesangial index, as previously shown [23,24,25]. However, in *eAdam17* and *tAdam17* deletion, diabetes-induced glomerular hypertrophy and mesangial matrix expansion were lost. These results suggested that tissue-specific deletion of *Adam17* from either endothelial cells or proximal tubular cells protected glomeruli from diabetic deleterious effects, probably by decreasing macrophage infiltration in the glomeruli of diabetic animals [26,27].

We and others demonstrated podocyte loss in diabetic mice [1,28,29]. In this line, we observed a decrease in podocyte number in the glomeruli of all diabetic mice. Interestingly, Guo et al. described TNF-α as a key mediator of HG-activated macrophages to induce podocyte apoptosis [27]. This may explain why we observed a decrease in podocyte loss in diabetic *tAdam17KO* mice. Blocking renal *Adam17* and consequently TNF-α signaling at a proximal tubular level might induce a tubular-glomerular feedback recovery that leads to podocyte protection during DN. In this line, Hasegawa et al. proved that molecular changes in proximal tubular cells affected contiguous cells including podocytes [30].

Serum ACE2 activity in diabetes has been widely described [1,31,32] and ADAM17 has been shown to induce this shedding [33]. Our study also showed increased circulating ACE2 activity in diabetic mice, which was reduced by *Adam17* deletion in proximal tubular cells. In concordance, Riera et al. demonstrated that paricalcitol administration in NOD mice significantly decreased circulating ACE2 activity in diabetic mice [11]. These results suggest a possible crosstalk between both proteins in which ADAM17 in the proximal tubular cell may transduce cellular signals somehow resulting in increased circulating ACE2.

In concordance with previous studies [1,31], we observed that diabetic mice presented increased kidney ACE2 activity that could be a compensatory beneficial mechanism, counterbalancing the deleterious effect of ANG II accumulation. Interestingly, after *eAdam17* deletion, a further increase in renal ACE2 activity was detected in diabetic mice in comparison with diabetic wild-type mice. These data suggested that because of diabetes, more ACE2 was produced at the tubular level and due to the deletion of *eAdam17*, less ACE2 shedding occurred from the endothelium, resulting in ACE2 accumulation in the renal cortex. In contrast, *tAdam17* deletion attenuated the increase of renal ACE2 activity in diabetic mice. We surmise that *tAdam17* deletion attenuates ACE2 shedding, leading to more ANG II degradation and therefore decreased activation of inflammatory signaling pathways in diabetic mice, preventing the increase in renal ACE2 activity.

ADAM17 upregulation leads to the activation of TNFR and EGFR signaling pathways, stimulating the shedding of its substrates and inducing a protein matrix accumulation, ending in renal inflammation and fibrosis [13,18,21]. We observed an increase in soluble TNF-α in diabetic wild-type mice that was reduced with a specific deletion of *Adam17*. These results suggested that blocking *Adam17* at a renal level during DN is enough to prevent TNF-α shedding and attenuate renal inflammation. Decreased renal *Tnf-α* and increased *Tnfr1* gene expression were found in diabetic *eAdam17KO* mice. These results may be due to a possible negative feedback, since the decrease in the release of TNF-α into the circulation could be well explained by the absence of ADAM17. In these circumstances, more TNFR1 may be needed to balance the downstream signaling pathway.

Omote et al. demonstrated increased renal macrophage infiltration in diabetic KK-Ay mice that was reduced by etanercept, a TNF-α inhibitor [34]. In line with these results, we observed increased macrophage infiltration in diabetic wild-type kidneys. Instead, both *eAdam17* and *tAdam17* deletion reduced F4/80 immunostaining in diabetic mice when compared with diabetic wild-type mice. Additionally, *eAdam17KO* mice were protected against the increase in *Ccl5* gene expression. The attenuation of inflammatory markers suggests that *Adam17* deletion ameliorates kidney inflammation by decreasing TNFR signaling.

ADAM17 releases the active ectodomains of EGFR ligands in injured proximal tubular cells. Accordingly, EGFR is persistently activated and induces the pro-inflammatory response favoring kidney fibrosis [16,35]. EGFR ligands are found to be low-affinity (TGF-α, epiregulin, and amphiregulin) or high-affinity EGFR ligands (HG-EGF). However, only low-affinity EGFR ligands can be responsible for sustained profibrotic EGFR activation [35]. In concordance, we observed higher renal *Tgfα* gene expression but not increased *Hb-egf* gene expression in diabetic mice.

EGFR hyperactivation induces renal fibrosis by stimulating TGF-β signaling [36]. Activation of the TGF-β signaling pathway favors DN progression through enhancing synthesis of collagen, fibronectin, and laminin and blocking matrix metalloproteinase-mediated extracellular matrix degradation [37]. EGFR transactivation has been demonstrated to be required for TGF-β upregulation by high-glucose [38]. In concordance, we also observed increased *Tgfβ* and *Fn* gene expression and increased TGF-β and pAkt protein expression in diabetic mice. Overstreet et al. showed that TGF-β protein expression in tubular epithelial cells was decreased after the administration of erlotinib, an EGFR inhibitor [39]. We observed that only *eAdam17* deletion prevents the activation of pAKT and the upregulation of *Tgf-β* gene expression in the diabetic renal cortex. However, both *eAdam17* and *tAdam17* deletion protects from TGF-β and collagen accumulation. In this line, immunofluorescence on HKC-8 spheroids showed increased accumulation of type-IV collagen when incubated in HG medium that was decreased with *Adam17* deletion. These findings suggest that in *Adam17*-deleted spheroids, EGFR activation is reduced due to less shedding of the EGFR substrates. Hence, TGF-β signaling is downregulated, leading to less type-IV collagen production.

In an experimental model of ischemia–reperfusion injury, Kefaloyianni et al. demonstrated that *tAdam17* deletion decreases α-SMA expression after kidney injury [35]. In this line, our results demonstrated that both *eAdam17* and *tAdam17* deletion prevent the accumulation of α-SMA induced by diabetes. Immunofluorescence on HKC-8 spheroids strengthen the fact that *tAdam17* deletion exerts a higher effect on blocking kidney fibrosis.

## 4. Materials and Methods

### 4.1. Animal Models and Diabetes Induction

Experiments were performed on wild-type and endothelial or proximal-tubular *Adam17KO* male mice on a C57BL/6 background. Mice were housed in ventilated cages with full access to chow and water. Diabetes was induced in 12-week-old mice by two intraperitoneal injections of 150 mg/kg streptozotocin (STZ) (Sigma, St Louis, MO, USA) on 4 h-fasted mice in 2 consecutive weeks as previously described [28]. Induction of diabetes was confirmed by measuring non-fasting blood glucose (BG) with the ACCU-CHEK Compact System (Roche, Basel, Switzerland) on blood samples from the caudal vein. Mice were considered diabetic when BG > 13.88 mmol/L during the first 4 weeks after STZ administration.

Diabetic mice were followed for 19 weeks after diabetes induction. At the end of the follow-up, mice were sacrificed by terminal surgery as previously described [28]. Blood was extracted by cardiac puncture and serum was obtained by centrifugation at 6000 g for 10 min. Mice were perfused with cold PBS prior to kidney removal. Half of the right kidney was embedded in OCT (Sakura Finetek, Alphen aan den Rijn, Netherlands) medium and the other half was maintained in 10% formalin solution (Sigma-Aldrich, Saint Louis, MO, USA) for paraffin embedding. The remaining tissue was snap frozen on liquid nitrogen and kept at −80 °C for further analyses.

### 4.2. Generation of the Specific Endothelial or Tubular Adam17KO Mice

We conditionally deleted Adam17 in endothelial (*eAdam17*) or renal proximal tubular (*tAdam17*) cells. *Adam17^flox/flox^* mice (kindly provided by Dr. Raines, Washington University, Saint Louis, MO, USA) [40], tamoxifen-inducible platelet-derived growth factor (Pdgf)-iCreER mice (kindly provided by Dr. Fruttiger, University College London, London, UK) [41] and spontaneous phosphoenolpyruvate carboxykinase (Pepck)-CreER mice (kindly provided by Dr. Haase, Vanderbilt University, Nashville, TN, USA) [42] were previously described. The inducible and spontaneous Cre recombinase was fused to the PDGF or PEPCK promoter, allowing the generation of *Adam17* deletion in endothelial or proximal tubular cells, respectively. The Pepck-CreER transgene contains a mutated version of the Pepck promoter, which reduces Pepck expression in the liver by 60% and increases Pepck expression in the kidney by 10-fold [43]. *Adam17^flox/flox^* mice presented two loxP sites surrounding the exon 5 of the *Adam17* gene. After recombination, the excision of the sequence results in a frame shift producing a nonsense protein [40] (Supplementary Figure S1). To induce *Adam17* gene recombination in endothelial cells, five intraperitoneal doses of 0.1 mg/g body weight of tamoxifen were administrated to 10-week-old mice. Wild-type (WT) mice receiving tamoxifen were used as controls. These animals did not carry any loxP site surrounding the exon 5 of the *Adam17* gene, so a recombination did not occur. Then, all WT mice expressed ADAM17 in all cell types.

### 4.3. Urine Albumin Creatinine Ratio

Urinary albumin excretion (UAE) was determined using the albumin-to-creatinine (ACR) ratio on morning spot urine collections obtained on the last week of the follow-up. Urinary albumin levels were measured by an ELISA kit (Albuwell M, Exocell, Philadelphia, PA, USA). Creatinine levels were measured by a colorimetric assay (Creatinine Companion, Exocell, Philadelphia, PA, USA). The albumin-to-creatinine ratio was calculated and expressed as µg Alb/mg Crea [11].

### 4.4. Immunohistochemistry on OCT-Embedded Tissue

Immunohistochemistry staining for ADAM17 was performed on 8µm-sections of OCT-embedded kidneys. Samples were fixed on 4% formalin (Sigma-Aldrich, Saint Louis, MO, USA) for 10 min. After PBS1X washing, slides were incubated in 3% H_2_O_2_ in methanol (MilliporeSigma, Burlington, MA, USA) for 10 min. To block non-specific interactions, slides were incubated with 10% goat serum (Sigma-Aldrich, Saint Louis, MO, USA) diluted in PBS1X for 1 h. Sections were incubated with an anti-ADAM17 antibody (ab2051; 1:100, Abcam, Cambridge, UK) for 1 h. After washing, the slides were incubated with HRP-conjugated anti-rabbit IgG (Agilent Technologies, Santa Clara, CA, USA) for 1 h. Arteries were microphotographed at ×200 magnification and kidney tubules at ×100 magnification.

### 4.5. Immunohistochemistry on Paraffined-Embedded Tissue

Paraffin-embedded tissues were cut into 3 µm sections, deparaffined in xylene, and rehydrated through graded alcohols. Sections were stained with periodic acid-Schiff (PAS) for glomerular area and mesangial matrix expansion measurements as previously reported [1]. Twenty microphotographs of glomeruli were taken at ×400 magnification for each animal.

Immunohistochemistry for β-galactosidase, podocyte marker Wilms Tumor 1 (WT-1), alpha smooth muscle actin (α-SMA), and F4/80 was also performed in sections of paraffin-embedded tissue. Antigen retrieval was carried out with 0.01 M sodium citrate buffer pH6 by heating in a pressure cooker. The slides were incubated in 3% H_2_O_2_ (Sigma-Aldrich, Saint Louis, MO, USA) in TBS1X for 15 min. Non-specific interaction blocking was done by 1% BSA (MilliporeSigma, Burlington, MA, USA) and 3% goat serum (Sigma-Aldrich, Saint Louis, MO, USA) for 1 h. Sections were then incubated with anti-β-galactosidase antibody (A11132, Invitrogen;1:2500, Carslbad, CA, USA), anti-WT-1 antibody (sc192; 1:1000, Santa Cruz Biotechnology, Dallas, TX, USA), anti-α-SMA antibody (A2547, mouse monoclonal, Sigma-Aldrich, Saint Louis, MO, USA; 1/1000 in PBS), or anti-F4/80 antibody (400501; 1:500, Biolegend, San Diego, CA, USA) overnight. After washing, the slides were incubated with HRP-conjugated anti-rabbit IgG, anti-mouse IgG or anti-rat IgG (Agilent Technologies, Santa Clara, CA, USA) for 1 h.

Binding of the antibodies was detected by oxidation of DAB using the Liquid DAB+Substrate Chromogen System (Dako, Santa Clara, CA, USA). Samples were counterstained with hematoxylin and dehydrated through graded alcohols and preserved with DPX mounting media (Sigma-Aldrich, Saint Louis, MO, USA). Twenty microphotographs of glomeruli stained with anti-WT-1 were taken at ×400 magnification. Six microphotographs of renal cortex stained with anti-α-SMA or anti-F4/80 were taken at ×100 or ×200 magnification respectively. All analyses were performed on ImageJ software v1.51j8 (Bethesda, MD, USA).

Picrosirius red staining was performed on 4.5 μm sections of paraffin-embedded kidneys. Tubulointerstitial collagen accumulation was semi-quantitatively measured (0–4 score) as previously described [44]. Representative images were taken at ×100 magnifications.

### 4.6. Western Blot

Kidney cortical tissue was prepared for immunoblot analysis with antibodies against phosphorylated and total Akt and TGF-β. Kidney cortex samples were homogenized in extraction buffer containing 50 mM HEPES, pH 7.4, 150 mM NaCl, 0.5% Triton X-100, 0.025 mM ZnCl_2_, (all from Sigma-Aldrich, Saint Louis, MO, USA) 0.1 mM Pefabloc SC Plus (Roche, Basel, Switzerland), EDTA-free protease inhibitor cocktail tablet (Roche, Basel, Switzerland), and phosphatase inhibitor cocktail (Sigma-Aldrich, Saint Louis, MO, USA). Protein concentration was determined using the Micro BCA Protein Assay Kit (ThermoFisher Scientific, Waltham, MA, USA).

Western Blot was performed by separating 15 µg of total protein in 7% SDS-polyacrylamide gels and transfer into Immobilon-P PVDF membranes (Millipore, Burlington, MA, USA). Membranes were incubated in skimmed milk blocking solution (5%) for 1 h and incubated overnight at 4 °C with anti-pAKT (Ser473) antibody (9271 S; 1:1000, Cell Signaling Technology, Danvers, MA, USA) in 2.5% BSA, anti-Akt antibody (9272 S; 1:2000, Cell Signaling Technology, Danvers, MA, USA) in 2.5% BSA and anti-TGFβ antibody (3711 S; 1:1000 Cell Signaling Technology, Danvers, MA, USA) in 2.5% BSA. HRP-conjugated anti-rabbit IgG antibody (A0545; 1:2000 or 1:4000, Sigma-Aldrich, Saint Louis, MO, USA) was used as a secondary antibody.

Proteins were detected in films (AGFA CURIX) after 3-min incubation with Clarity Western ECL Substrate (Bio-Rad, Hercules, CA, USA). Protein bands were quantified by densitometry with the ImageJ software v1.51j8 (Bethesda, MD, USA).

### 4.7. Soluble TNF-α ELISA

Serum TNF-α levels were measured using the Mouse TNF-α Quantikine ELISA Kit (R&D Systems, Minneapolis, MN, USA) according to the manufacturer’s instructions. Next, 50 μL of mouse serum were incubated with the Assay Diluent for 2 h in microplates coated with a monoclonal antibody specific for mouse TNF-α. After washing unbound substances, an enzyme-linked polyclonal antibody specific for mouse TNF-α conjugated to HRP was added and incubated for 2 h. After washing, the plate was incubated with the Substrate Solution for 30 min. The enzyme reaction yields a blue product that turns yellow when the Stop Solution was added. Finally, optical density was determined using the Tecan Infinite 200 reader at 450 nm and 570 nm for wavelength correction. Results were expressed as pg/mL.

### 4.8. 3D Cell Culture Set up and CRISPR/Cas9 Adam17 Silencing

Human proximal tubular cells (HKC-8) kindly provided by Dr. Nugraha were cultured in DMEM/F12 without glucose medium (Biowest LLC, Riverside, MO, USA) and were supplemented with low glucose (5.5 mM, Sigma-Aldrich, Saint Louis, MO, USA), 2.5% FBS (Biowest LLC, Riverside, MO, USA), 1% Insulin-Transferrin-Selenium (Corning, Corning, NY, USA), Penicillin (100 Units/mL), and Streptomycin (100 µg/mL) (Biowest LLC, Riverside, MO, USA) at 37 °C and 5% CO_2_.

To obtain the HKC-8 spheroids, the 3D Life dextran hydrogel kit (BioCat, Heidelberg, Germany) was functionalized with RGD peptide (BioCat, Heidelberg, Germany). The HKC-8 single cell suspension (around 57,600 cells) was mixed with the RGD-functionalized dextran hydrogel and crosslinked with PEG-based crosslinker as previously described [45].

Thirteen days post-seeding, the spheroids were incubated with either of the final concentrations of 35 mM d-glucose (HG, high-glucose), 5.5 mM d-glucose (LG, low-glucose), or 35 mM mannitol (M) (Sigma-Aldrich, Saint Louis, MO, USA) as an osmotic control for 72 h.

*Adam17* deletion on HKC-8 cells was performed using the human TACE CRISPR/Cas9 KO plasmid (sc-400827, Santa Cruz Biotechnology, Dallas, TX, USA). The TACE CRISPR/Cas9 KO Plasmid consists of a pool of three plasmids, each encoding for a GFP protein, the Cas9 nuclease, and a target-specific 20 nt guide RNA designed for maximum knockout efficiency.

Positive transfected cells were selected by GFP+ sorting. Cells were resuspended in PBS + 1% FBS and filtered directly before sorting using cell strainer caps in 5 mL FACS tubes (Corning, Corning, NY, USA). Non-transfected cells were used as controls to estimate background signals. The cell sorting was performed using the FACSAria III sorter (BD Biosciences, San Jose, CA, USA). GFP+ cells were collected in 5 mL tubes with complete DMEM-F12 medium and centrifuged at 300 g for 5 min. After aspiration, cells were resuspended in complete DMEM-F12 medium and seeded in the single well of a 24-well plate.

### 4.9. Immunofluorescence in Tubular Spheroids

Immunofluorescence for fibrotic markers such as type-IV collagen and α-SMA was performed on HKC-8 spheroids after fixing them in 4% PFA (ThermoFischer Scientific, Waltham, MA, USA) for 30 min. The spheroids were then permeabilized and blocked with 0.2% saponin (Sigma-Aldrich, Saint Louis, MO, USA), 3% BSA (Sigma-Aldrich, Saint Louis, MO, USA) and 20% FCS (Sigma-Aldrich, Saint Louis, MO, USA) for 40 min and incubated with a primary α-SMA antibody (A5228; 1:1000, Sigma-Aldrich, Saint Louis, MO, USA) and a type-IV Collagen antibody (C1926; 1:1000, Sigma-Aldrich, Saint Louis, MO, USA) for 1 h. After secondary antibodies, anti-mouse IgG (Alexa 488, A32723; 1:500, ThermoFisher Scientific, Waltham, MA, USA) incubation for 1 h, samples were mounted with Fluorsave (Merck, Darmstadt, Germany) to minimize laser-induced photo bleaching. The quality of the established 3D-cell culture with mature HKC-8 spheroids was assessed by AQP-1 (SC-32737; 1:1000, Santa Cruz Biotechnology; Dallas, TX, USA) and GLUT-1 (Ab652; 1:500, Abcam; Cambridge, UK) staining.

Microscopical images from the middle of the spheroid core were acquired using a HC APO CS2 20x/0.75 IMM on a Leica SP8 inverted confocal microscope (Leica, Wetzlar, Germany). The 3D image stack was reconstructed using Imaris Software (Bitplane, Zürich, Switzerland). The experiment was repeated three times and eight to ten images were taken from each slide. The staining intensity was normalized to the spheroid volume.

### 4.10. Gene Expression

Renal cortex RNA was isolated from frozen tissue using the Tripure Isolation Reagent (Sigma-Aldrich, Saint Louis, MO, USA) as previously reported [23] and 1.5 µg of total RNA were retrotranscribed using the High Capacity cDNA RT Kit (ThermoFisher Scientific, Waltham, MA, USA). Gene expressions for *Adam17*, Angiotensin II receptor type 1 A (*At1ra*), Angiotensin II receptor type 2 (At2r), angiotensinogen (*Agt*), *Tnfα*, *Tnfr1*, fibronectin (*Fn*), *Tgfα*, heparin-binding EGF-like growth factor (*Hb-egf*), *Tgfβ*, and chemokine (C-C motif) ligand 5 (*Ccl5*) were determined by Real-Time PCR using LightCycler^®^480 SYBR Green I Master Mix (Roche, Basel, Switzerland). Glyceraldehyde-3-phosphate dehydrogenase (Gapdh) was used as a housekeeping gene. Primer sequences were synthesized by Sigma (Saint Louis, MO, USA) and described in Supplementary Table S1.

### 4.11. ACE2 Enzymatic Activity

The ACE2 fluorescent enzymatic assay was performed as previously described using the ACE2 quenched fluorogenic substrate Mca-Ala-Pro-Lys(Dnp)-OH (Enzo Biochem, Farmingdale, NY, USA) [11]. For ACE2 enzymatic activity, 2 µL of serum or 1 µg of total protein were incubated in duplicate with an ACE2 assay buffer consisting of 100 mM Tris-HCl, 600 mM NaCl, 10 µM ZnCl_2_ pH 7.5 (Sigma-Aldrich, Saint Louis, MO, USA) plus inhibitors: 100 µM captopril, 5 µM amastatin, 5 µM bestatin (all from Sigma-Aldrich, Saint Louis, MO, USA), and 10 µM Z-Prolyl-prolinal (Enzo Biochem, Farmingdale, NY, USA). The reaction was initiated with the addition of 10 µM (serum) or 5 µM (kidney) of fluorogenic substrate and incubated at 37 °C for 16 h or 4 h, respectively. Fluorescence was measured using the Tecan Infinite 200 (Männedorf, Switzerland) at λex320 nm/λem400 nm. Results were expressed as RFU/µL/h and RFU/µg/h, respectively.

### 4.12. Statistical Analyses

Statistical analyses between groups were performed by one-way ANOVA test (SPSS 22.0 software, Armonk, NY, USA). Non-parametric Kruskal–Wallis tests were performed between groups. Non-parametric Mann–Whitney tests were used for group-to-group comparisons. Data were expressed as mean ± SD. Significance was defined as *p* < 0.05.

## 5. Conclusions

In conclusion, this study shows that tissue-specific *Adam17* deletion protects against renal inflammation and fibrosis. Diabetic mice lacking specific *eAdam17* displayed attenuated renal inflammation and ameliorated renal fibrosis. *Adam17* deletion in the renal proximal tubular cells of diabetic mice attenuated podocyte loss, RAS, renal inflammation and fibrosis in terms of macrophage infiltration, collagen and α-SMA accumulation. The study of fibrotic markers on a renal proximal tubular 3D in vitro cell culture reinforced the animal findings. All things considered, these results suggest that *Adam17* should be considered as a therapeutic strategy for treating DN.

## Figures and Tables

**Table 1:** Blood glucose, body weight, and KW/BW ratio were measured after 19 weeks of diabetes. Fasting blood glucose was determined during the follow-up and at the end of the study, body weight and KW/BW ratio were recorded in all experimental groups. Values are expressed as mean ± SD.

Endothelial ADAM17 Model				
	WT-CONT	WT-DB	KO-CONT	KO-DB
Fasting blood glucose (mg/dL)	175.44 ± 20.26	430.29 ± 31.49 *	185.30 ± 15.68	485.9 ± 86.91 *
Body weight (g)	34.61 ± 5.37	26.35 ± 3.61 *	33.09 ± 3.34	24.16 ± 2.70 *
KW/BW	0.97 ± 0.19	1.45 ± 0.31 *	1.00 ± 0.11	1.41 ± 0.16 *
**Proximal Tubular ADAM17 Model**				
	WT-CONT	WT-DB	KO-CONT	KO-DB
Fasting blood glucose (mg/dL)	177.13 ± 6.45	397.33 ± 17.89 *	202.67 ± 5.92	362.58 ± 27.54 *
Body weight (g)	34.38 ± 4.45	28.35 ± 3.27 *	34.94 ± 4.40	27.87 ± 2.15 *
KW/BW	1.07 ± 0.17	1.35 ± 0.15 *	1.00 ± 0.10	1.25 ± 0.20 *^$^

* *p* ≤ 0.05 DB vs NoDB; ^$^ *p* ≤ 0.05 KO vs WT.

## Data Availability

Not applicable.

## Supplementary Materials

The following are available online at https://www.mdpi.com/article/10.3390/ijms22115520/s1, Figure S1: Confirmation of *Adam17* deletion on endothelial and renal proximal tubular cells. (a) Schematic representation of the targeting construct, showing replacement of the loxP sites for Cre-mediated recombination on the *Adam17* and *LacZ* genes. (b) Endothelial and proximal tubular *Adam17* deletion was confirmed by immunohistochemistry. Images were taken at ×20 magnifications for the arteries and at ×10 magnifications for the renal cortex. (c) β-galactosidase staining as control of recombination for proximal tubular *Adam17* deletion. Images were taken at 10× magnifications. (d) *Adam17* gene expression from *tAdam17* model; n = 7–8 in each group. * *p* < 0.05 DB vs. CONT, $ *p* < 0.05 KO vs. WT. Figure S2: Influence of diabetes and *Adam17* deletion on ACR ratio. (a) ACR ratio from *eAdam17* model. (b) ACR ratio from *tAdam17* model. ACR, albumin-to-creatinine ratio; CONT, control; DB, diabetic; eAdam17, endothelial ADAM17; KO, knockout; tAdam17, proximal tubular ADAM17; WT, wild-type. * *p* < 0.05 DB vs. NoDB; n = 6–10 in each group. Figure S3: Influence of diabetes and *Adam17* deletion on *Angiotensinogen* gene expression. (a) *Agt* gene expression from *eAdam17* model. (b) *Agt* gene expression from *tAdam17* model. Agt, angiotensinogen; CONT, control; DB, diabetic; eAdam17, endothelial ADAM17; KO, knockout; tAdam17, proximal tubular ADAM17; WT, wild-type. * *p* < 0.05 DB vs. NoDB, $ *p* < 0.05 KO vs. WT; n = 7–8 in each group. Figure S4: Influence of diabetes and *Adam17* deletion on *Hb-egf* gene expression. (a) *Hb-egf* gene expression from *eAdam17* model. (b) *Hb-egf* gene expression from *tAdam17* model. CONT, control; DB, diabetic; eAdam17, endothelial ADAM17; KO, knockout; tAdam17, proximal tubular ADAM17; WT, wild-type; n = 7–8 in each group. Table S1: Primer sequences used for Real Time qPCR analysis.
