# Supplementary material for: Both Specific Endothelial and Proximal Tubular Adam17 Deletion Protect against Diabetic Nephropathy
Source: Int J Mol Sci. 2021 May 24;22(11):5520. doi: 10.3390/ijms22115520 (PMC8197223; doi:10.3390/ijms22115520)
Supplement: Supplementary file 1 [file ijms-22-05520-s001.zip › ijms-1211420-SI.pdf]

Supplementary Figure 1

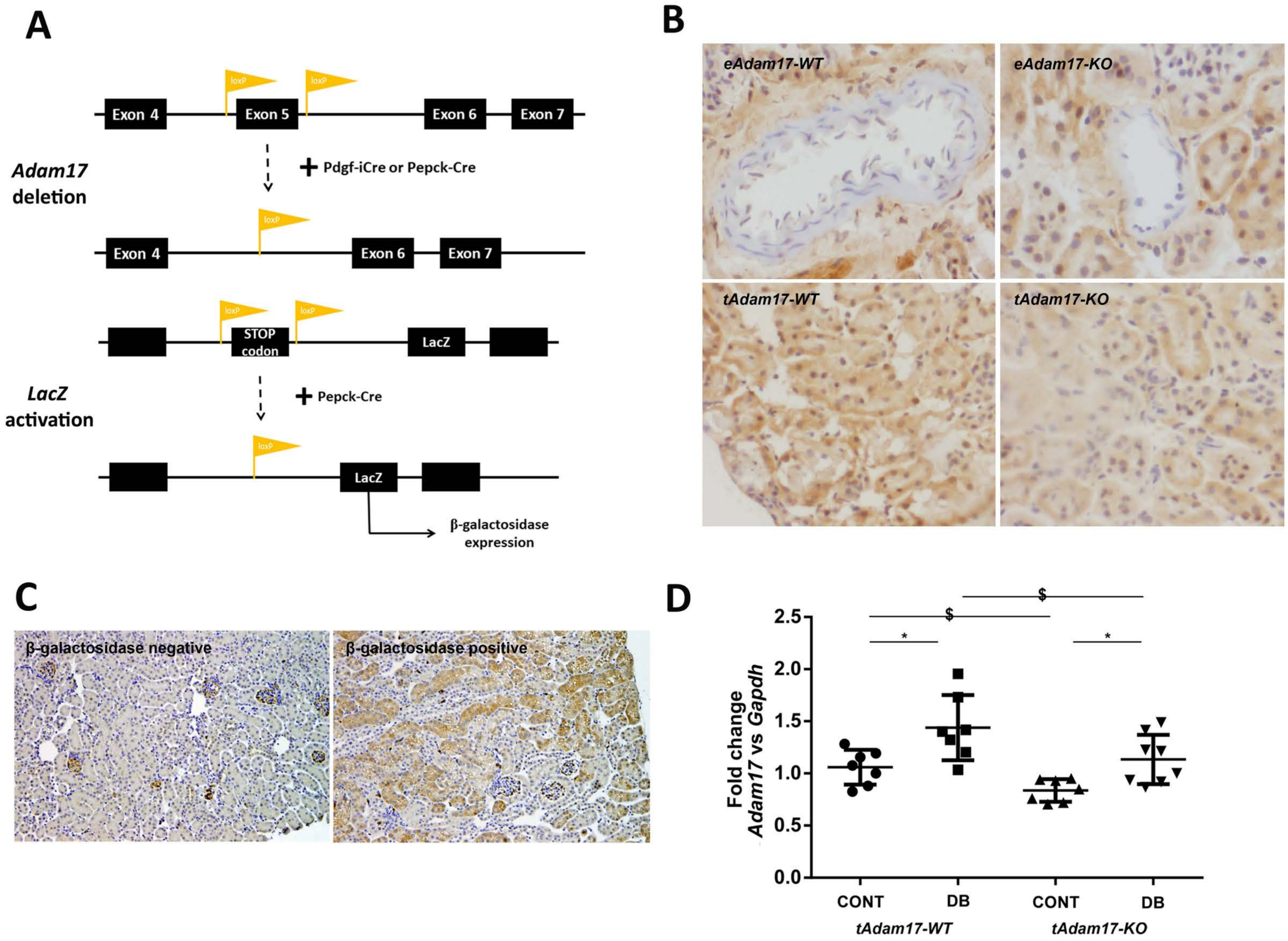

Supplementary Figure 2

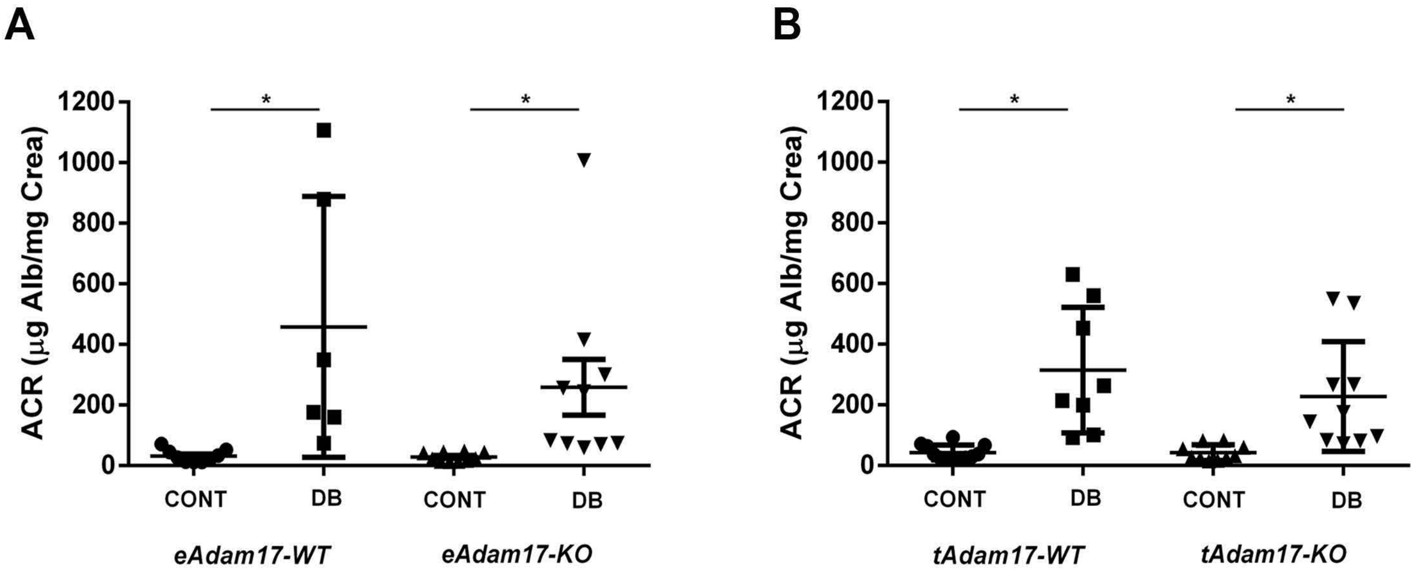

Supplementary Figure 3

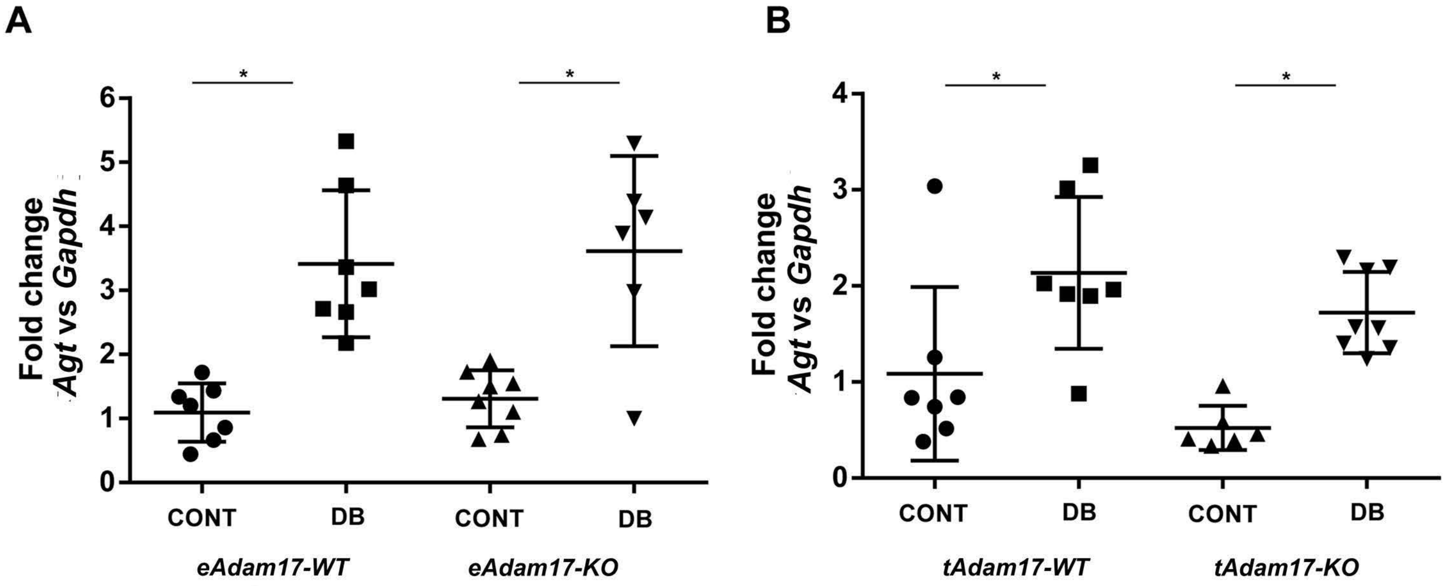

Supplementary Figure 4

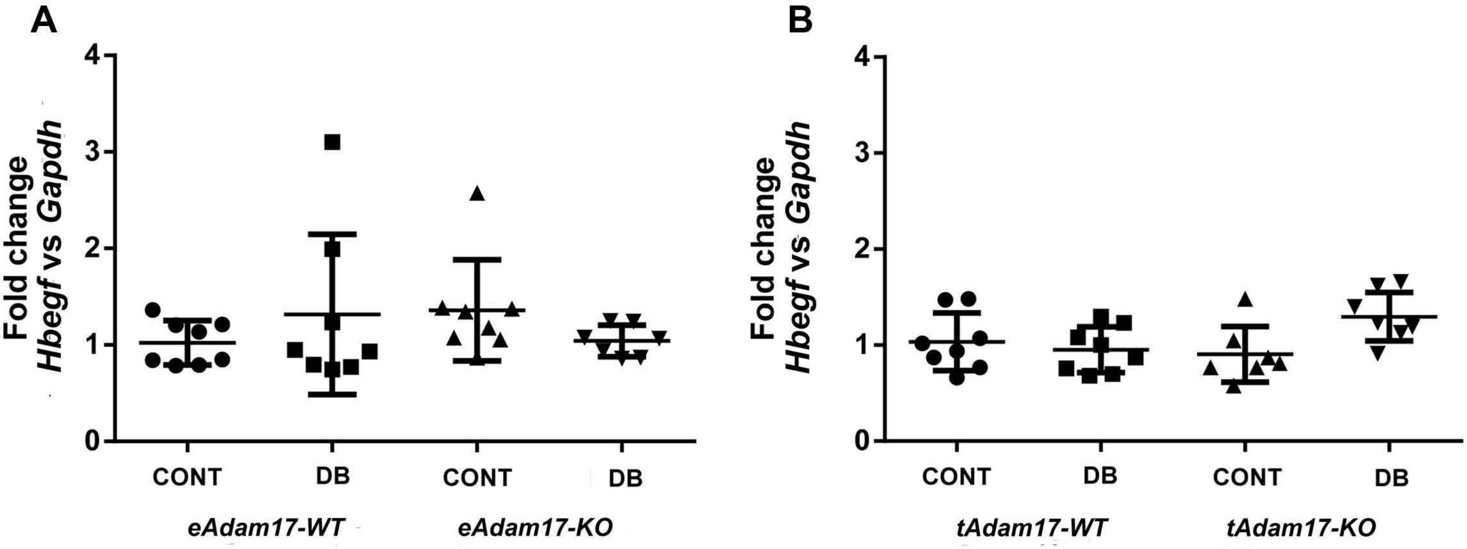

Supplementary Table 1. Primer sequences used for Real Time qPCR analysis

	FORWARD	REVERSE
**AT1Ra**	5’-CAAAGCTTGCTGGCAATGTA-3’	5’-ACTGGTCCTTTGGTCGTGAG-3’
**AT2R**	5’-TAATCAGCCTAGCCATTGGTTT-3’	5’-TGTTCTCGGGGGAGTAAGTAAA-3’
**Agt**	5’-CGTGCCCCTAGGTGAGAGAG-3’	5’-TCCAAGTCAGGAGGTCGTTC-3’
**TNFα**	5’-GACTAGCCAGGAGGGAGAACAG-3’	5’-CAGTGAGTGAAAGGGACAGAACCT-3’
**TNFR1**	5’-ACGAATCACTCTGCTCCGTG-3’	5’-TCGCAAGGTCTGCATTGTCA-3’
**FN**	5’-GCCACCGGAGTCTTTACTACC-3’	5’-TCTCTGTCACCTCGGTGTTG-3’
**TGFα**	5’-AGGAAGAGAAGCCAGCATGT-3’	5’-GCAGTGATGGCTTGCTTCTT-3’
**HB-EGF**	5’-TGGTGGCTGTAGTACTGTCGTC-3’	5’-TCATAACCTCCTCTCCTGTGGT-3’
**TGFβ**	5’-CAACAACGCCATCTATGAGAAA-3’	5’-CTTCCCGAATGTCTGACGTATT-3’
**CCL5**	5’-CTGCTGCTTTGCCTACCTCT-3’	5’-GTGACAAACACGACTGCAAGAT-3’
**GAPDH**	5’-TCA TTG ACC TCA ACT ACA TGG TCT-3’	5’-CTT GAC TGT GCC GTT GAA TTT-3’
